# Impact of Cereal Production Displacement from Urban Expansion on Ecosystem Service Values in China: Based on Three Cropland Supplement Strategies

**DOI:** 10.3390/ijerph19084563

**Published:** 2022-04-10

**Authors:** Siyu Sheng, Bohan Yang, Bing Kuang

**Affiliations:** 1 College of Public Administration, Central China Normal University, Wuhan 430079, China; shengsiyu@mails.ccnu.edu.cn (S.S.); kuang117@ccnu.edu.cn (B.K.); 2 Institute of Nature Resources Governance, Central China Normal University, Wuhan 430079, China

**Keywords:** cropland displacement, urban expansion, land-use modeling, food security, ecosystem service values

## Abstract

The acceleration of global urban expansion constantly occupies high-quality cropland and affects regional food security. The implementation of cropland protection policies has alleviated the pressure of cropland loss worldwide, and thus keeping a dynamic balance of cereal production. Such a displacement of cereal production from the lost cropland to the supplemented cropland has resulted in the massive losses of natural habitats (such as forests, grasslands, and wetlands) as well as ecosystem service values. However, the impact of cereal production displacement caused by different cropland supplement strategies has not been concerned. Therefore, taking China (mainland) as a case, this study used the LANDSCAPE model to simulate cereal production displacement caused by urban expansion and cropland supplement between 2020 and 2040, based on three scales of the Chinese administration system (i.e., the national level, the provincial level, and the municipal level). The natural habitat loss and corresponding ecosystem service value (ESV) loss were assessed. The results show that the national-scale cereal displacement will lead to a large reclamation of cropland in North China, causing the most natural habitat loss (5090 km^2^), and the least ESV loss (46.53 billion yuan). Cereal production displacement at the provincial and municipal scales will lead to fewer natural habitat losses (4696 km^2^ and 4954 km^2^, respectively), but more ESV losses (54.16 billion yuan and 54.02 billion yuan, respectively). Based on the national food security and ecological conservation in China, this study discussed the reasons for the ecological effects of cereal production displacement, direct and indirect natural habitat loss of urban expansion, and cropland protection policies in China. We suggest that China’s cropland protection policy should emphasize avoiding large-scale cropland displacement and occupation of natural habitat with high ESV for cropland supplement.

## 1. Introduction

In the last few decades, rapid urban expansion has become a major driving force of global cropland loss [1,2,3]. From 1985 to 2015, global urban land was increased from 3.63 × 10^5^ km^2^ to 6.53 × 10^5^ km^2^, which took 1.85 × 10^5^ km^2^ of cropland and a large amount of crop production [4]. Meanwhile, cropland expanded into forests, grasslands, and wetlands around the world to meet the growing demands for food to sustain fast-growing human populations [5,6]. From 2001 to 2019, the global cropland area had a net increase of 3.23 × 10^5^ km^2^ (FAOSTAT, 2021). The loss and supplement of cropland led to the spatial movement of cereal production, namely cereal production displacement [7]. Thus, the cereal production displacement is always accompanied by the spatial movements of cropland. Recent studies indicated that cropland or cereal production displacement has caused the losses of natural habitat [8,9], ecosystem service values [10,11,12,13], and the increases of environmental risk globally [14,15].

Similar to cropland change, the losses and gains of cereal production differ in regions. Urban expansion mainly occurs in developed areas, such as East Asia, Europe, and North America, where a large amount of high-quality cropland was occupied [4,16]. Meanwhile, the supplement of cereal production is more likely to take place in South America, Southeast Asia, Africa, as well as areas with an under-developed economy and higher-level agricultural modernization, such as Nigeria and Brazil [8,10,17]. In addition, urban expansion not only occupies fertile cropland resources but also causes serious cereal production losses [7,18,19]. Nevertheless, cropland supplement is mainly derived from areas with fragile ecosystems and poor farming conditions [20,21,22,23]. To meet food demand for the increasing population and changing diet, natural habitats need to be cultivated into new cropland to supplement the lost cereal production, which may cause more ecological problems [16].

Land endowment, affluence, population migration, and other factors influence cereal production displacement among countries. For example, under the strict cropland protection policies, Chinese grain production has been self-sufficient for decades [24,25]. However, due to its large population and unbalanced plantation structure, China still needs to massively import soybean and other oil crops from other countries [26]. Restricted by the climate condition and cropland resources, some developed countries face a deficit in domestic agricultural production and generally import from developing countries [27,28]. Global warming has altered the heat conditions in high latitudes for cereal production supplements, such as Canada and Russia [29,30]. Meanwhile, due to the superior farming conditions, rich cropland resources, and cheaper labor force, some tropical areas such as Amazon Plain, sub-Sahara Africa, and Southeast Asia have more significant expansion in cropland and increase in cereal production than other regions [31,32,33].

With the development of the spatially explicit model on land-use change, many studies have explored the transitions of cereal production displacement by combining the land-use change models and the crop yield estimation models. For example, van Vliet et al. [7] projected that between 2000 and 2040, urban growth globally would cause a displacement of almost 65 Mton of cereal production. Zheng et al. [34] explored the impact of cropland supplements on the trade-offs between cereal production and ecosystem services by combining the LANDSCAPE model with the GAEZ model. In addition, many studies also focus on simulating the impact of future cropland change on cereal production or ecosystem services [16,17,35], the changes of cereal production and ecosystem service value triggered by urban expansion [36,37], and the direct and indirect loss of natural habitat from cropland displacement [8,9,13]. However, the impact of cereal production displacement through different cropland supplement strategies on the ecosystem has not been of concern. 

This study takes the Chinese Mainland as a case, to simulate cereal production displacement at three Chinese administrative levels (i.e., the national level, the provincial level, and the municipal level) from 2020 to 2040 by using the LANDSCAPE model, and to assess corresponding changes in ecosystem service values. From the perspective of regional cropland protection strategy, exploring the impacts of cereal production displacement on ecosystem is of important significance to alleviate the conflict between food security and ecosystem conservation.

## 2. Data and Methods

### 2.1. The LANDSCAPE Model

LANDSCAPE (LAND System Cellular Automata Model for Potential Effects) is a spatially explicit model for land-use change simulation based on the Cellular Automata Model [38]. This study uses the model to simulate cereal production displacement respectively under three levels of China’s administrative boundaries for the period 2020–2040. The LANDSCAPE model simulates land-use changes with two key features: a hierarchical allocation strategy and the possibility of assigning changes in multiple land-use classes. It considers conversion probabilities between various land-use classes and reveals the dynamic simulation and optimal configuration of land-use changes through the hierarchical allocation strategy [38]. 

Hierarchical allocation strategy: The LANDSCAPE model reflects the evolutionary characteristics of different land-use classes, dividing them into active and passive categories. Active land-use classes, such as construction land and cropland, refer to the change directly caused by human activities, including the land-use classes with the direct demand of human production and life. The passive land-use classes, such as wetland, forest, and grassland, are not directly determined by human needs, but due to the change of active land-use classes.

Conversion probability: The conversion probability of a grid cell is determined by its suitability and resistance. Specifically, suitability refers to the driving force of the grid cell converting from one land-use class to the others, and resistance refers to the difficulty meanwhile. The great conversion probability indicates the land-use class of the grid cell is more likely to convert to other land-use classes. When active land-use classes grow, passive ones are occupied by active ones according to their suitability and resistance. The conversion probability is calculated by:

(1) $$CP_{l,tu}=\frac{S_{l,tu}}{R_{l,cu}}$$

where *CP_l,tu_* is the probability of location *l* to convert to target land-use class *tu*, *S_l,tu_* is the suitability of location *l* to convert to target land-use class *tu*, and *R_l,cu_* represents the resistance of location *l* to convert from current land-use class *cu* to other land-use classes. *S_l,tu_* is determined by constraints on location, neighbor, biophysical, and socioeconomic parameters. *R_l,cu_* depends on the current land-use class *cu* and its resistance. In this paper, the resistance coefficients of each land-use class refer to previous studies as shown in Table 1. The calculation formula for *S_l,tu_* is:

(2) $$S_{l,tu}=(1+{\left(1+(-\ln r)\right)}^{\alpha })\times PG_{l,tu}\times Con\left(C_{l,tu}\right)\times \Omega_{l,tu}$$

where *r* is a random number within 0–1, *α* is an integer within 1–10 to control the size of the random variable. *PG_l,tu_* represents the impacts of parameters, such as elevation, slope, GDP, population density, and other parameters. *Con (C_l,tu_)* represents conversion constraints. In this study, rivers, rural settlements, and Nature Reserves are limited to convert. Ω*_l,tu_* is the neighbor conversion probability of each land-use class. 

**Figure 1:**
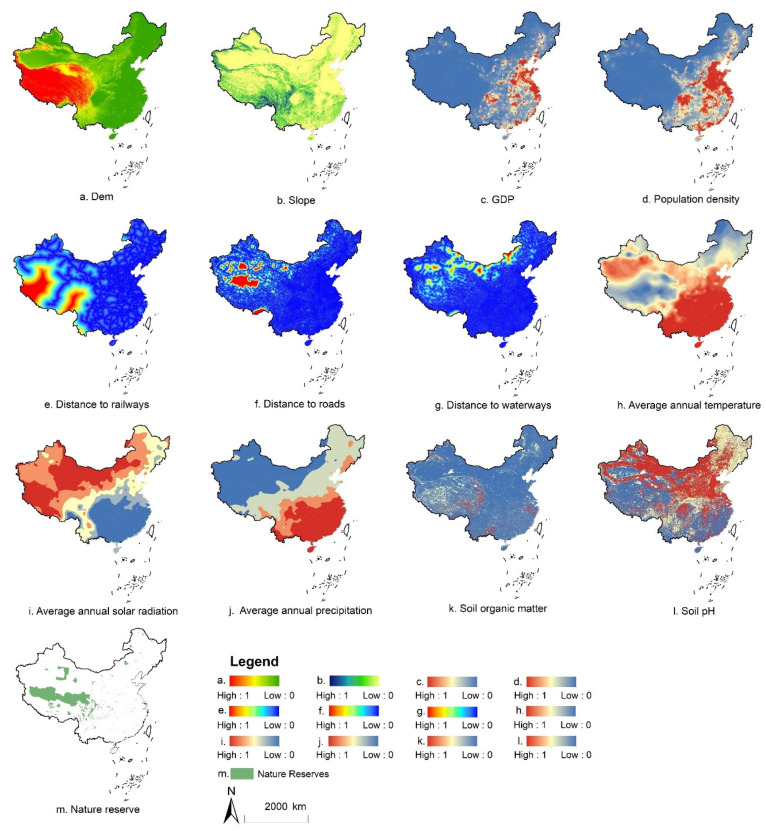
The parameters used for calculating conversion probability.

This study uses the Random Forest model to calculate the *PG_l,tu_*. The process can be described as follows: Firstly, the land-use data and its influencing factors are randomly sampled to obtain a sample point data required for excavating conversion rules. Secondly, the cell conversion rules are obtained from the sampled data by the Random Forest algorithm. Finally, the conversion probabilities of all grid cells for each land-use class are prepared. The parameters used in this process are shown in Figure 1.

### 2.2. Model Calibration

Simulation accuracy can be evaluated by comparing the differences between the simulated and the observed land-use maps in 2020. The confusion matrix is a commonly used method for accuracy evaluation. However, it fails to reflect the difference between changing and unchanged cells during land-use evolution, and may cause an overestimation of model accuracy. To avoid this, van Vliet et al. [39] developed the Kappa Simulation Index, which can evaluate the accuracy of land-use change simulation more objectively and clearly. Thus, this study uses the Kappa Simulation Index to evaluate the simulation accuracy of LANDSCAPE model. 

The Kappa Simulation score holds values ranging from −1 to 1, where 1 indicates a perfect agreement, and 0 indicates that the agreement is only as good as a random distribution of given class transitions. A negative Kappa Simulation score demonstrates a lower accuracy, while a positive value can be interpreted as being more accurate than a random distribution, and a score closer to 1 indicates higher confidence.

### 2.3. Design of Cropland Supplement Strategies

Cereal production displacement is the result of spatial movement of cropland which is lost somewhere and supplemented elsewhere. Accordingly, cereal production displacement can be decomposed into two steps. The first is cereal production loss as cropland is occupied by urban land, and the second is equivalent cereal production supplemented from new cropland converted from natural habitats such as forest, grassland, wetland, and unused land. On the basis of a same spatial distribution of cropland loss from urban expansion, we considered three levels of China’s administration system (i.e., nation, province, and municipality) as the boundaries of cropland supplements, to compensate for the lost cereal production within the whole study area.

Specifically, three cropland supplement strategies were designed as (1) the National Scale (*SN*): no boundary limitations for cereal production supplement. After the cereal production is lost by urban expansion, the equivalent cereal production could be supplemented anywhere in China’s mainland. (2) Provincial Scale (*SP*): the lost and supplemented cereal production must be balanced for each province. (3) Municipal Scale (*SM*): the lost and supplemented cereal production must be balanced for each municipality. Thus, the process of cereal production loss caused by urban expansion is the same for each scenario; however, the process of cereal production supplement is different. This paper uses the Markov Chain model by Matlab to predict the national demand for urban land in 2040, to simulate urban expansion for the year 2020–2040. Land-use maps for 2000, 2010, and 2020 are used for the prediction that China’s urban land will increase by 10.82% and reach 78,866 km^2^ until 2040.

### 2.4. Evaluation of ESV Changes

Costanza et al. [40] proposed the global ecosystem service value assessment model and it has been widely applied in the world. Xie et al. [41,42] improved this model based on expert knowledge method, that is, considering six ecosystems (forest, grassland, cropland, wetland, water, and unused land) and nine service types to build the equivalent factor table. Based on the research of Xie et al. [41], and the net profit of grain production per unit area of cropland ecosystem in 2010 [42,43], this paper estimates the service value of each ecosystem per unit area (Table 2).

Therefore, based on the ecosystem service value per unit area, the ecosystem service value of each land-use class can be calculated. The formula is as follows:

(3) $$ESV=\sum \left(A_{k\;}\times VC_{k}\right)$$

where *ESV* refers to ecosystem service value, *A_k_* is the area of land-use class *k*, and *VC_k_* is the *k* ecosystem service value. Moreover, the losses of ecosystem service value in this study only considered the ecosystems of forest, grassland, wetland, and unused land. 

### 2.5. Data Resource

This study mainly uses five datasets. The first is land-use data for 2000, 2010, and 2020, derived from the Resource and Environment Science and Data Center, Chinese Academy of Sciences, Beijing, China (RESDC, https://www.resdc.cn (accessed on 20 March 2021)) with a spatial resolution of 1 km. Among them, cropland refers to the land where crops are planted, including paddy fields and dry land; forest refers to growing trees, shrubs, and other forestry lands; grassland refers to all kinds of grassland mainly growing herbs with coverage of more than 5%; wetland refers to lakes, tidal flats, and ponds except for rivers; urban land refers to built-up areas for cities, counties, and towns; rural settlement refers to residential land for rural living; unused land refers to the currently unused land, such as desert, saline-alkali soil, marsh, bare land, and other land that is difficult to use.

**Figure 2:**
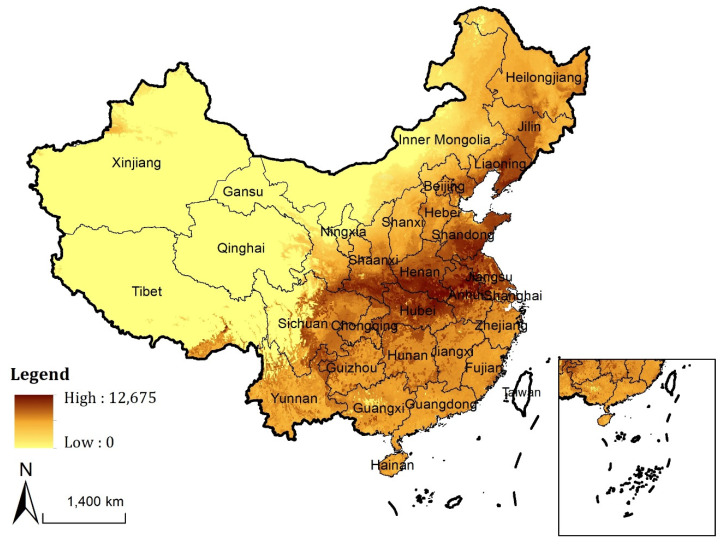
Spatial distribution of potential cereal productivity in China (Unit: kg/ha).

The second is datasets of agro-ecological attainable yield, derived from GAEZ products (https://gaez.fao.org (accessed on 15 April 2021)) for the period 1981–2010 for wheat, maize, and paddy rice under rain-fed for all phase conditions, high input level, and with CO_2_ fertilization using climate data source CRUTS32 based on historical data. We mixed the three main crops of China (accounting for about 97.7% of total Chinese grain production) to generate the data of potential cereal productivity, and resample the data from 5 arc-minutes of original resolution to 1 km (Figure 2).

The third is the administrative boundary data, including three administrative scales at the national, provincial, and municipal levels, all obtained from the National Administrative Division Database of RESDC. Among them, the provincial data includes 31 provinces (excluding Hong Kong, Macao, and Taiwan), the municipal data includes 339 municipalities.

The fourth datasets are the parameters to calculate conversion probability for the LANDSCAPE model, including socio-economic data, meteorological data, terrain data, soil data, traffic data, nature reserve data.

The fifth dataset is the population statistic data, used to predict the demand for urban construction land in 2040 (Table 3).

## 3. Results

### 3.1. Model Validation

This study simulated the land-use change from 2010 to 2020 to evaluate the accuracy of the LANDSCAPE model. The accuracy was calculated through the comparison among the observed land-use map in 2010 and 2020 and the simulated land-use map in 2020 (Table 4). The Kappa Simulation values are greater than 0 for all land-use classes, which indicate that the model is accurate enough to simulate the land-use in 2040. Specifically, the relatively high *K_simulation* values for urban land, rural settlements, cropland, and unused land represent that the LANDSCAPE model has high accuracy in simulating these land-use classes.

### 3.2. Cropland Change from 2020 to 2040

**Figure 3:**
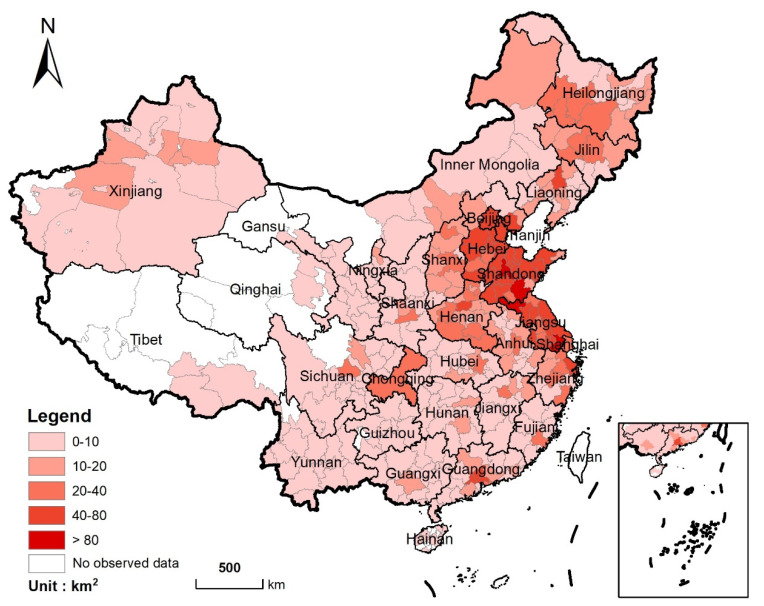
Spatial distribution of cropland loss from 2020 to 2040 (Statistics at the municipal level).

From 2020 to 2040, the nationwide urban areas will be expanded by 7700 km^2^ at a rate of 385 km^2^ per year. Approximately 61% (4701 km^2^) of the new urban land is developed by taking cropland. The cropland loss will occur mainly in the east coast and central regions of China (Figure 3). Among them, the most significant loss of cropland will be concentrated in Shandong and Jiangsu provinces (823 km^2^ and 770 km^2^, respectively), accounting for 17.5% and 16.4% of the total cropland loss, respectively. In contrast, the cropland loss in Tibet, Qinghai, and Hainan will be less than 10 km^2^, respectively, due to their unobvious urban expansion.

**Figure 4:**
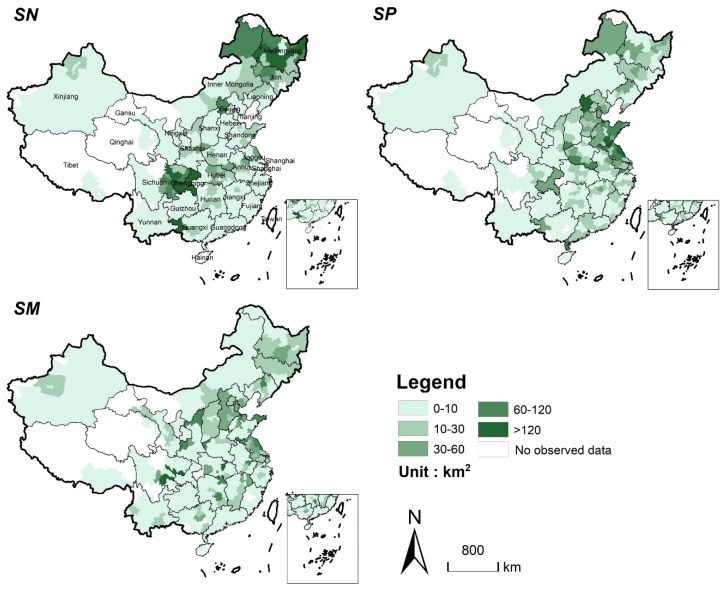
Spatial distribution of cropland supplement in the three scenarios from 2020 to 2040. (Statistics at the municipal level. *SN*: Cereal production balanced for the nation; *SP*: Cereal production balanced for each province; *SM*: Cereal production balanced for each municipality).

At the same time, large amounts of new cropland will be supplemented from natural habitats for cereal production supplement. However, the quantity and spatial location of new cropland are different due to the different cropland supplement strategies. Figure 4 shows the spatial distribution of new cropland in scenarios *SN*, *SP*, and *SM*. From 2020 to 2040, the total amount of supplemented cropland will reach 5090 km^2^, 4696 km^2^, and 4954 km^2^ in *SN*, *SP*, and *SM*, respectively. In *SN*, the new cropland will be mainly concentrated in the Northeast Plain and Sichuan Basin area, where are also rich in cropland reserve resources. In *SP* and *SM*, the new cropland will be mainly distributed in the areas with serious cropland loss, such as central and eastern regions.

### 3.3. Cereal Production Displacement Caused by Urban Expansion

**Figure 5:**
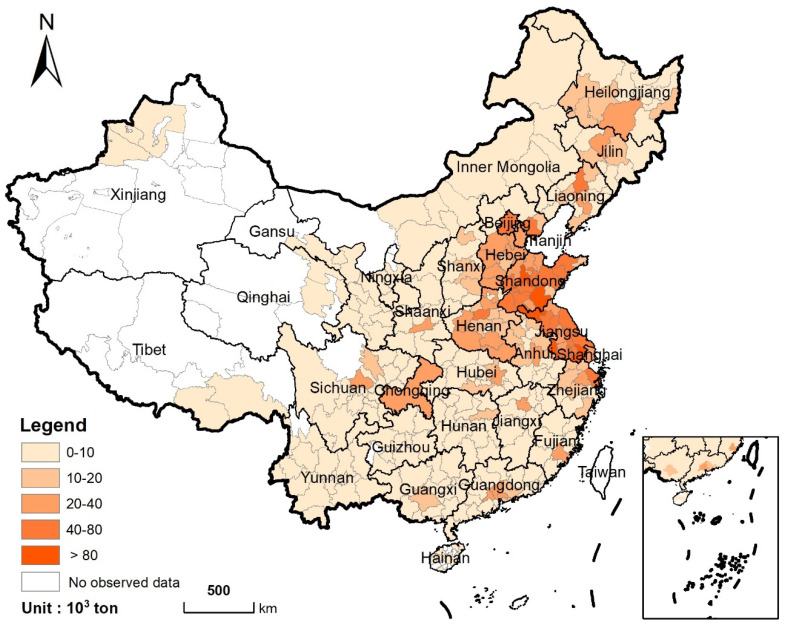
Spatial distribution of cereal production loss from 2020 to 2040 (Statistics at the municipal level).

Between 2020 and 2040, the urban expansion will cause a total loss of 3.838 Mton in cereal production by occupying cropland. It is notable that the cereal production loss will be more prominent in major grain-producing areas of China, such as the provinces of Henan, Shandong, Jiangsu, Hebei in central and eastern China, and provinces of Liaoning and Heilongjiang in northeast China. These are also the areas with rapid urbanization or high-quality cropland (Figure 5). Among them, Shandong and Jiangsu are the two provinces with the largest cereal production losses (0.77 Mton and 0.75 Mton will be lost, respectively), accounting for 20.1% and 18.5% of the total cereal production losses, respectively. On the contrary, the loss of cereal production in northwest China will be lower, due to small cropland loss and limited potential cereal productivity.

**Figure 6:**
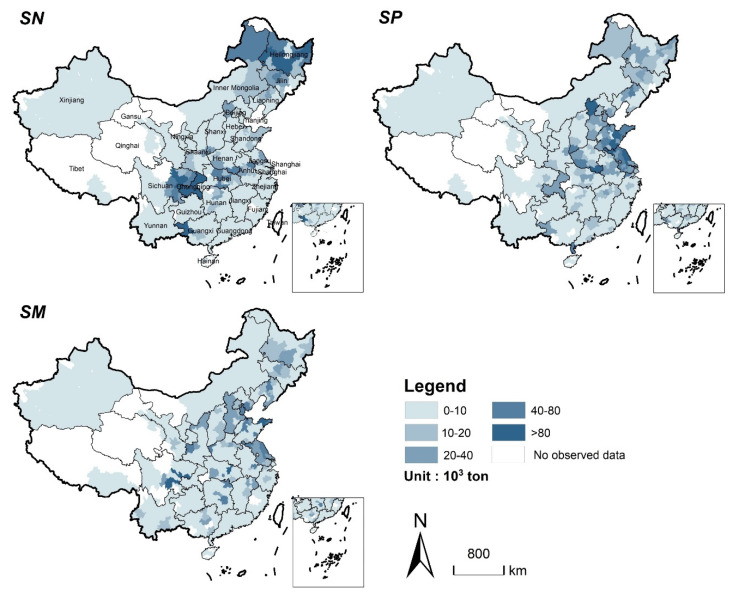
Spatial distribution of cereal production supplement in the three scenarios from 2020 to 2040 (Statistics at the municipal level).

The supplemented cropland, respectively, will bring 3.838 Mton, 3.842 Mton, and 3.862 Mton of cereal production in scenarios *SN*, *SP*, and *SM*, which indicates all cropland supplement strategies achieve a cereal production balance in lost and supplemented cropland nationwide. In spite of this, the amounts and locations of supplement cropland are different for each strategy due to the spatial heterogeneity of the potential cereal productivity. In *SN*, cereal production will be mainly supplemented in Central China, Sichuan Basin, and the Northeast Plain (Figure 6). The supplements of cereal production in Sichuan and Heilongjiang provinces accounted for more than half of the total supplements (1.11 Mton and 0.96 Mton will be supplemented, respectively). In *SP* and *SM*, the new cropland will be mainly distributed in central and eastern China, especially in Shandong and Jiangsu provinces, which are also the areas with more serious losses of cereal production. 

### 3.4. ESV Losses Caused by Production Displacement

**Figure 7:**
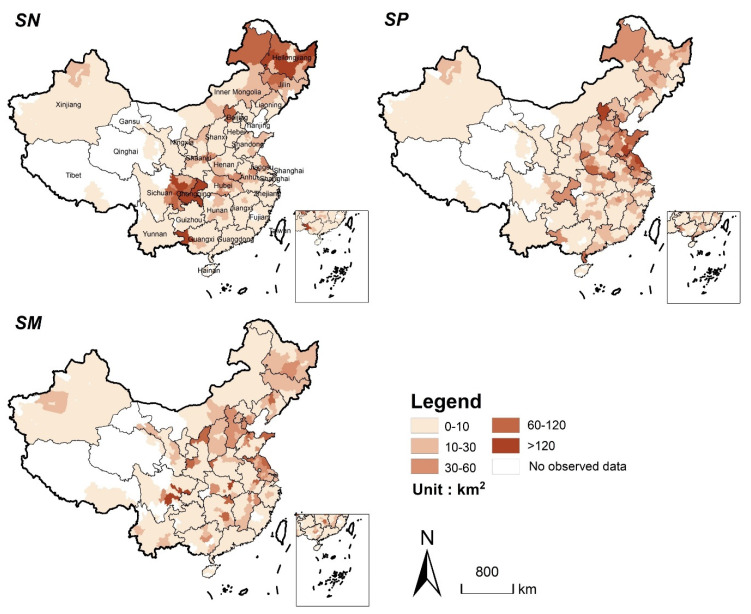
Spatial distribution of natural habitat loss caused by cereal production displacement in the three scenarios from 2020 to 2040 (Statistics at the municipal level).

To supplement the lost cereal production caused by urban expansion, a large number of natural habitats are converted into cropland. From 2020 to 2040, 5069 km^2^, 4696 km^2^, and 4954 km^2^ of natural habitats will be lost in *SN*, *SP*, and *SM*, respectively. In addition, different cropland supplement strategies resulted in a significant difference in the structures and the spatial distributions of natural habitat loss. In *SN*, the main sources of cereal production supplement are forests and grasslands, while in *SP* and *SM*, the main sources are wetlands (Table 5). Figure 7 shows the spatial distribution of natural habitat loss for each scenario. In *SN*, the supplement of cereal production will mainly come from Sichuan Basin and Northeast Plain. Among them, the provinces of Sichuan and Heilongjiang will lose 1388 km^2^ and 1454 km^2^ of natural habitats, respectively, accounting for 27.3% and 28.5% of the total natural habitat losses, respectively. In *SP*, the most significant loss of natural habitat will be located in central and eastern China. Specifically, during the study period, the losses of natural habitat in the Jiangsu and Shandong provinces will account for 33.86% of the total losses. Similarly, the losses of natural habitat in the Sichuan Basin, the North China Plain, and the eastern coastal areas will be more obvious in *SM*.

**Figure 8:**
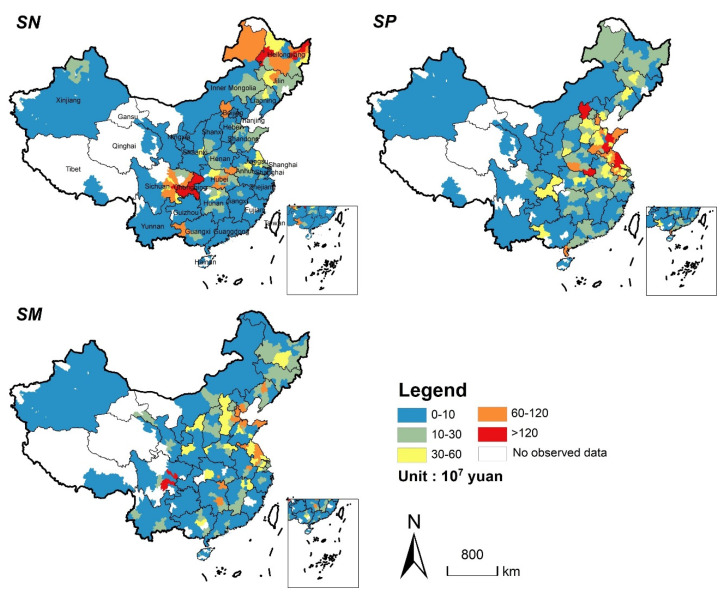
Spatial distribution of ESV loss caused by cereal production displacement in the three scenarios from 2020 to 2040 (Statistics at the municipal level).

From 2020 to 2040, the total ESV losses from the cereal production displacement of three scenarios will reach 46.53 billion yuan, 54.16 billion yuan, and 54.02 billion yuan, respectively. We count the ESV losses of three scenarios to a unified scale (the municipal scale), which is clearer to reflect the spatial distribution (Figure 8). In *SN*, the ESV losses will be more significant in Sichuan Basin and Northeast Plain. Especially, the losses of Sichuan and Heilongjiang will account for almost half of the total (they will lose 13.51 billion yuan and 8.83 billion yuan, respectively) (Table 6). In *SP*, the ESV losses will be mainly concentrated in the central and eastern regions, such as Jiangsu and Shandong provinces, which will lose 12.68 billion yuan and 10.26 billion yuan, respectively. Additionally, the substantial reduction in ESV in *SP* is mainly caused by the wetland loss due to its relatively high ecosystem service value. In *SM*, it is mainly manifested by the losses of forest and wetland in the Sichuan Basin and the central and eastern regions, resulting in a significant reduction of ESV in the changing areas.

## 4. Discussion

### 4.1. Impact of Cereal Production Displacement on ESV

This paper simulated China’s cereal production displacement caused by urban expansion from 2020 to 2040 and assessed the changes of ESV under three cropland supplement strategies. In this period, Chinese urban expansion will lead to a loss of 4701 km^2^ cropland with 3.838 Mton cereal production. In scenario *SN*, cereal production displacement with the characteristic of “gain in north and loss in south, gain in west and loss in east” is similar to the pattern of cropland change. This keeps the variation tendency of China’s cropland resource as well as cereal production in the past few decades [36,44]. A possible reason is that the growing temperature and precipitation conditions promote the crop planting in the north [45,46]. New cropland in the north mainly comes from the reclamation of grassland and unused land, while that in southwest China mainly comes from deforestation. However, in *SP* and *SM*, the supplemented cropland is mainly generated from wetlands, due to the abundant water resources in the east and south of China. When liberalizing the provincial boundary, cereal production displacement will cause the most natural habitat loss, but the lowest ESV loss, because cropland supplement in *SN* mainly comes from grassland and unused land, which have lower ESVs. 

### 4.2. Direct and Indirect Impacts of Urban Expansion on Ecosystem Service Values

**Figure 9:**
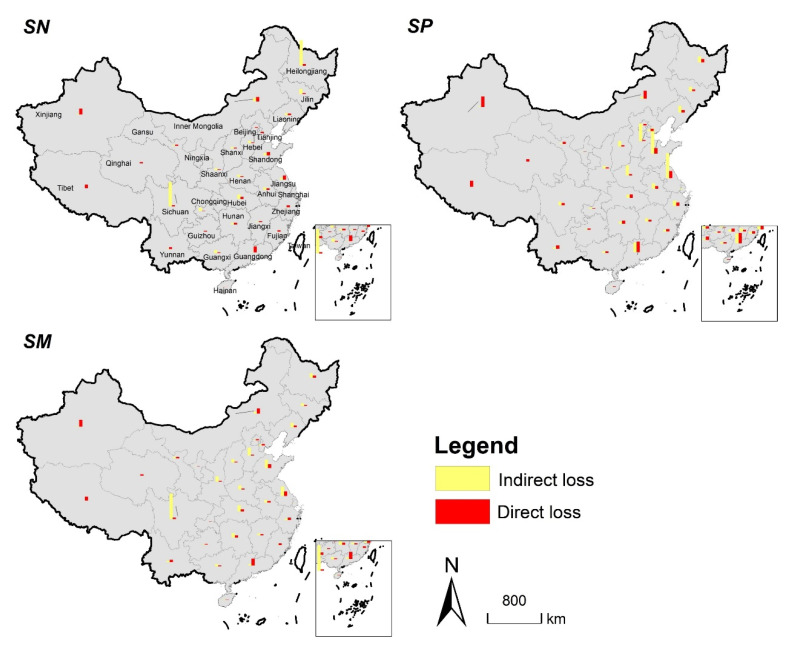
Spatial distribution of direct and indirect losses of natural habitat in three scenarios from 2020 to 2040 (Statistics at the provincial level).

Urban expansion mainly occupies circumjacent cropland and natural habitat, causing the direct loss of natural habitat through occupying. Furthermore, cropland displacement will cause indirect loss of natural habitat [9], which is mainly manifested in the conversion of natural habitat into cropland to compensate for the cropland loss caused by urban expansion. Our study found that all three scenarios showed that indirect loss of natural habitat is 50% more than the direct loss. Figure 9 shows the spatial distribution of direct and indirect losses under different scenarios. Similar research at the global scale [8] and the local scale [9,13] also agrees that the indirect loss of natural habitat should not be neglected. Furthermore, it is necessary to evaluate the indirect impact of urban expansion on ecosystem when considering the cropland protection policies. Thus, more attention should be paid to exploring the pathways of land management to coordinate cropland protection and ecosystem conservation.

### 4.3. Policy Implication for Cropland Protection

Cropland protection policies and measures have been applied worldwide, such as agricultural land zoning protection [47,48], land-use control system [49], agricultural subsidies [50,51,52], and basic cropland protection system [53]. For national food security, the Chinese government has implemented a series of strict cropland protection policies in the past 40 years [36,54]. Among them, the “requisition-compensation balance of cropland policy” (RCBC, which is proposed in 1997) is always the core policy of the whole cropland protection system. The latest adjustment of RCBC in 2018 mainly changed intra-provincial balance to national balance, that is, cropland can be supplemented across provinces. However, our results show that cross-provincial cropland supplement would result in the greatest negative impacts on natural habitat at the national scale. From the existing studies, the northern cropland expansion has no significant contribution to local socio-economic development, and the grain produced goes mainly to the south and the population continues to move south [55,56]. This phenomenon may be the opposite to other regions of the world. Brazil and Argentina and other developing countries have improved their economic development by developing export-oriented agriculture [57,58]. Additionally, cropland expansion has moved Brazilian farmers to eastern Paraguay and eastern Bolivia, which have no ban on deforestation [59]. In addition, our results also show that the implementation of cereal production balance within the province will lead to the most loss of ecosystem service value. Therefore, we suggest that China should try to avoid the large-scale cereal production displacement and the occupation of natural habitat with high ESV when supplementing cropland as well as cereal production. In addition, more attention should be paid to the improvement of existing cropland quality to reduce the conflict between food security and ecosystem conservation.

### 4.4. Uncertainty

This study provides a reference for the implementation of cropland protection policy and alleviating the conflict between food security and ecological conservation in China. However, there are still some limitations. First, the spatial resolution of the grid data adopted in this study is relatively rough, which is not suitable for the experiment at the county level of China. Datasets with smaller resolutions may help to provide more accurate performance in future work. Second, we assumed that the cereal potential productivity remained consistent during 2020–2040. In fact, the cereal potential productivity varies over time due to the changes of climate, soil, water resource, etc. [29,60]. In addition, cereal production potential can be supplemented by two pathways, namely cropland cultivation and the improvement intensification of existing cropland; this study only considered the former. 

## 5. Conclusions

This paper simulated cereal production displacement at three Chinese administrative levels from 2020 to 2040 by using the LANDSCAPE model and assessed corresponding changes in ecosystem service values based on the unit area value equivalent method. The results show that the urban expansion will cause a loss of 3.838 Mton of cereal production by occupying cropland. However, the cereal production displacement produced different ecological effects in different scenarios. Scenario *SN* will lose the most natural habitats with 5069 km^2^ totally, and the *SP* and *SM* will lose less with 4696 km^2^ and 4954 km^2^, respectively, indicating that liberalizing the provincial boundary of cereal production displacement will lead to more natural habitat loss. *SN* lost the least ESV (46.53 billion yuan), while the most ESV will be lost in *SP* (54.16 billion yuan), followed by *SM* (54.02 billion yuan). These findings have some implications for cropland protection policies as well as their ecological effects. In addition to cereal production displacement, assessing the ecological consequences caused by cropland intensification is also worthy of concern.

## Figures and Tables

**Table 1:** Resistances of each land-use classes.

Land-Use Class	Cropland	Forest	Grassland	River	Wetland	Urban Land	Rural Settlement	Unused Land
Resistance	1	1.25	1.25	1.5	1.25	1.5	1.5	1

**Table 2:** Ecosystem service values per unit area in China (Yuan/hm^2^).

Primary Classification	Secondary Classification	Ecosystem of Cropland	Ecosystem of Forest	Ecosystem of Grassland	Ecosystem of Wetland	Ecosystem of Water	Ecosystem of Unused Land
Supply service	Food production	3406.50	1124.15	1464.80	1226.34	1805.45	68.13
	Raw material production	1328.54	10,151.37	1226.34	817.56	1192.28	136.26
Regulation service	Gas regulation	2452.68	14,716.08	5109.75	8209.67	1737.32	204.39
	Climate regulation	3304.31	13,864.46	5314.14	46,158.08	7017.39	442.85
	Hydrology regulation	2623.01	13,932.59	5177.88	45,783.36	63,940.01	238.46
	Waste treatment	4735.04	5859.18	4496.58	49,053.60	50,586.53	885.69
Support service	Soil conservation	5007.56	13,694.13	7630.56	6778.94	1396.67	579.11
	Biodiversity	3474.63	15,363.32	6370.16	12,569.99	11,684.30	1362.60
Culture service	Aesthetic landscape	579.11	7085.52	2963.66	15,976.49	15,124.86	817.56
total		26,911.38	95,790.8	39,753.87	186,574	154,484.8	4735.05

**Table 3:** Datasets’ sources and descriptions.

Datasets	Data Source	Data Description
Land-use data	RESDC	Land-use map in 2000 is used to project urban land demand of 2040 Land-use map in 2010 is used to simulate the land use of 2020 Land-use map in 2020 is used for model calibration
Administrative boundary data	RESDC	The national boundary data are used for scenario *SN* The provincial boundary data are used for scenario *SP* The municipal boundary data are used for scenario *SM*
Cereal production potential data	GAEZ	Cereal production potential dataset is used as restricted condition of cereal production displacement in LANDSCAPE model
Meteorological data	China Meteorological Administration	Data of average annual precipitation in 2018 are used to calculate the conversion probability Data of average annual accumulated temperature in 2018 are used to calculate the conversion probability Data of average annual solar radiation in 2018 are used to calculate the conversion probability
Terrain data	The Shuttle Radar Topography Mission (SRTM)	DEM data are used to calculate the conversion probability Slope data extracted from DEM are used to calculate the conversion probability
Soil data	Harmonized World Soil Database (HWSD)	Soil type is used to calculate the conversion probability Soil organic carbon is used to calculate the conversion probability Soil PH value is used to calculate the conversion probability
Traffic data	Open Street Map	Euclidean distance to roads of 2020 is used to calculate the conversion probability Euclidean distance to railways of 2020 is used to calculate the conversion probability Euclidean distance to waterways of 2020 is used to calculate the conversion probability
Population statistic data	RESDC	Total population of the Chinese mainland in 2015 is used to project urban land demand of 2040
GDP	RESDC	The spatial distribution of GDP of China in 2015 is used to calculate the transfer probabilities
Nature reserve data	RESDC	Restricted development zones for urban expansion and cropland supplement

**Table 4:** Fine assessment of land-use simulation results (2010–2020).

	Cropland	Forest	Grassland	River	Urban Land	Rural Settlement	Unused Land
*K_simulation*	0.261	0.107	0.052	0.214	0.547	0.277	0.303
*K_Transloc*	0.470	0.327	0.433	0.454	0.587	0.313	0.434
*K_Transition*	0.555	0.328	0.119	0.472	0.931	0.886	0.697

**Table 5:** The loss of different natural habitats caused by cereal production displacement in the three scenarios from 2020 to 2040 (unit: km^2^).

Natural Habitat Loss	Natural Habitat	*SN*	*SP*	*SM*
	Forest	2587	1725	2467
	Grassland	1182	1040	927
	Wetland	903	1792	1428
	Unused land	418	139	132
	Total	5090	4696	4954

**Table 6:** Losses of natural habitat and ESV caused by cereal production displacement in the three scenarios from 2020 to 2040.

Province	*SN*		*SP*		*SM*	
	Natural Habitat Loss/km^2^	ESV Loss/ 10^7^ Yuan	Natural Habitat Loss/km^2^	ESV Loss/ 10^7^ Yuan	Natural Habitat Loss/km^2^	ESV Loss/ 10^7^ Yuan
Anhui	138	193.55	168	234.71	112	153.14
Beijing	1	0.96	24	25.63	18	17.16
Chongqing	184	180.19	38	35.97	1	0.96
Fujian	4	2.71	55	51.19	8	7.8
Gansu	39	19.77	23	12.85	67	40.48
Guangdong	12	11.63	308	323.03	73	85.76
Guangxi	174	139.97	49	36.85	105	94.18
Guizhou	8	5.98	17	11.24	18	14.44
Hainan	8	8.01	9	9.88	17	18.1
Hebei	123	118.89	557	501.47	370	442.42
Heilongjiang	1454	883.85	189	111.4	212	135.25
Henan	125	150.62	319	357.27	189	186.36
Hubei	255	379.3	102	155.24	306	415.71
Hunan	83	108.9	69	88.79	208	248.05
Inner Mongolia	188	126.2	57	36.53	86	53.37
Jiangsu	102	178.43	785	1268.19	462	756.98
Jiangxi	33	41.6	100	121.61	76	86.69
Jilin	283	242.86	132	114.02	130	119.31
Liaoning	108	120.53	215	214.29	215	218.07
Ningxia	4	2.71	6	3.51	8	6.89
Qinghai	2	0.8	4	1.59	1	0.96
Shaanxi	96	71.74	77	54.26	253	149.98
Shandong	134	193.4	805	1025.56	402	522.69
Shanghai	0	0	22	27.56	11	13.05
Shanxi	75	56.48	171	128.72	160	119.67
Sichuan	1388	1351.22	110	109.54	1229	1236.79
Tianjin	3	5.6	49	82.34	52	84.09
Tibet	1	0.96	1	0.96	1	0.96
Xinjiang	30	17.45	20	8.51	21	12.69
Yunnan	21	15.77	46	35.02	68	56.23
Zhejiang	14	22.49	169	228.08	75	103.96
Total	5090	4652.55	4696	5415.82	4954	5402.2

## Data Availability

Not applicable.
